# Supplementary figures and images for: 3,3’-Diindolylmethane induces apoptosis and autophagy in fission yeast
Source: PLoS One. 2021 Dec 10;16(12):e0255758. doi: 10.1371/journal.pone.0255758 (PMC8664220; doi:10.1371/journal.pone.0255758)

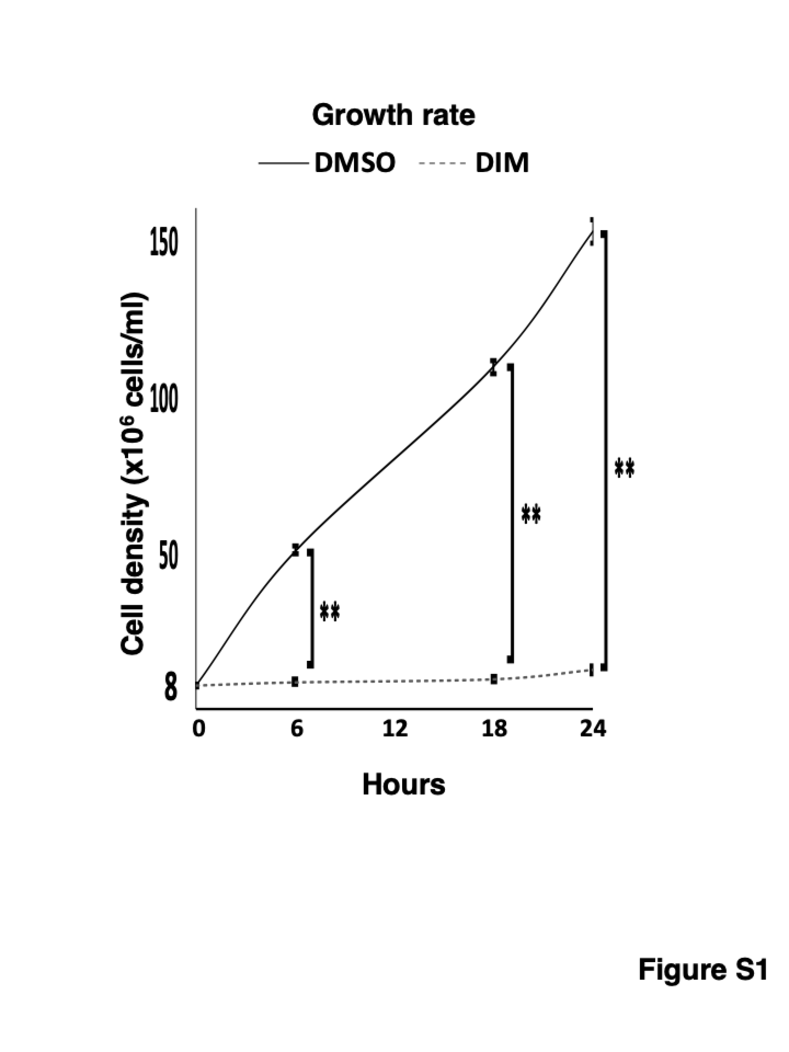

Supplement: S1 Fig — 20 μg/ml 3,3’-Diindolylmethane (DIM) was added to the log-phase cells and the cell growth was measured during the first day. (TIF) [file pone.0255758.s001.tif]

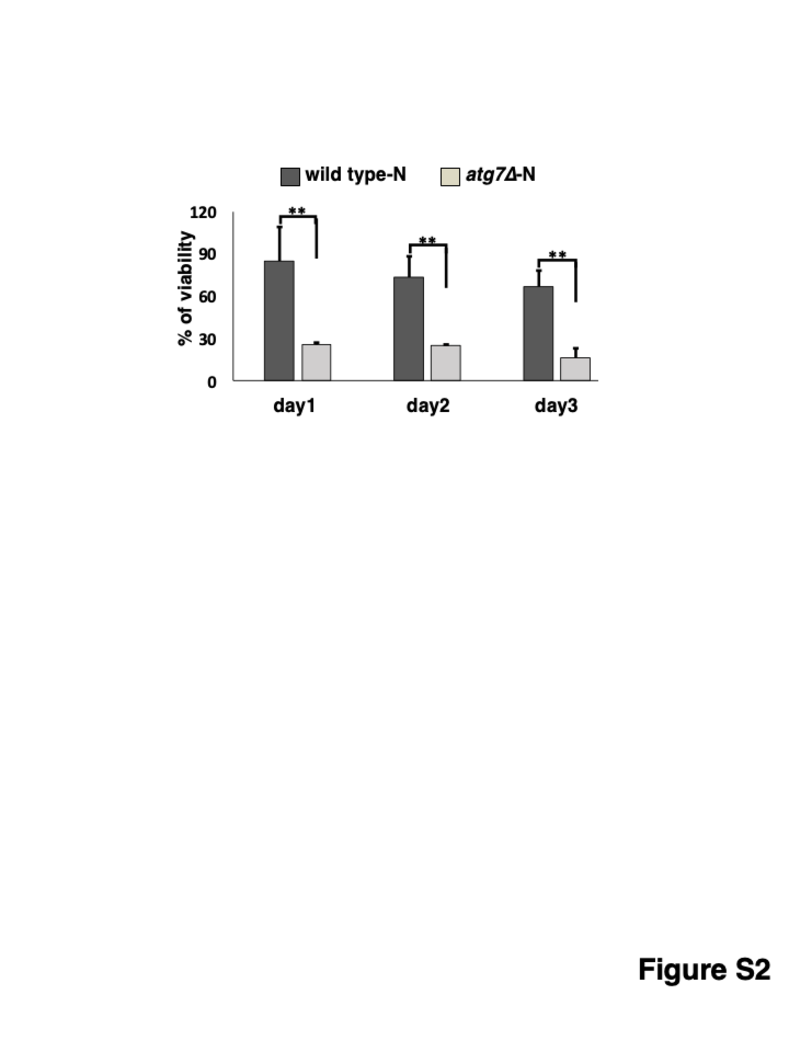

Supplement: S2 Fig — Percentages of viability under nitrogen starvation for three days are shown. The log-phase cells of auxotrophic wild type strain (h90 ade6-216 leu1-32, ura4-D18, lys1-131) and its atg7Δ mutant were cultured in YEA medium. Then cells were cultured in EMM without any nitrogen source and lysine, uracil, adenine, and leucine. Day1 means that cells are cultured under nitrogen starvation for one day. (TIF) [file pone.0255758.s002.tif]

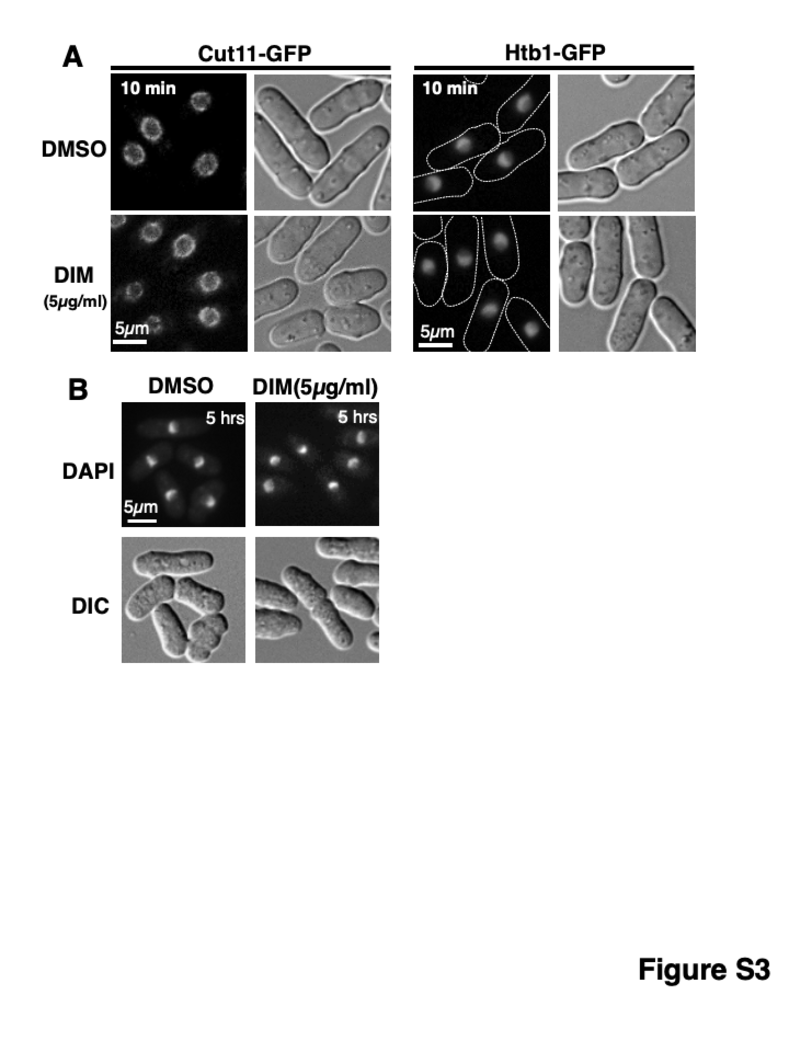

Supplement: S3 Fig — (A) 5 μg/ml 3,3’-Diindolylmethane (DIM) was added to the log-phase cells expressing Cut11-GFP and Htb1-GFP and assayed after 10 minutes (B) DAPI staining was performed for log-phase cells after 5 hours of incubation with 5 μg/ml DIM. (TIF) [file pone.0255758.s003.tif]

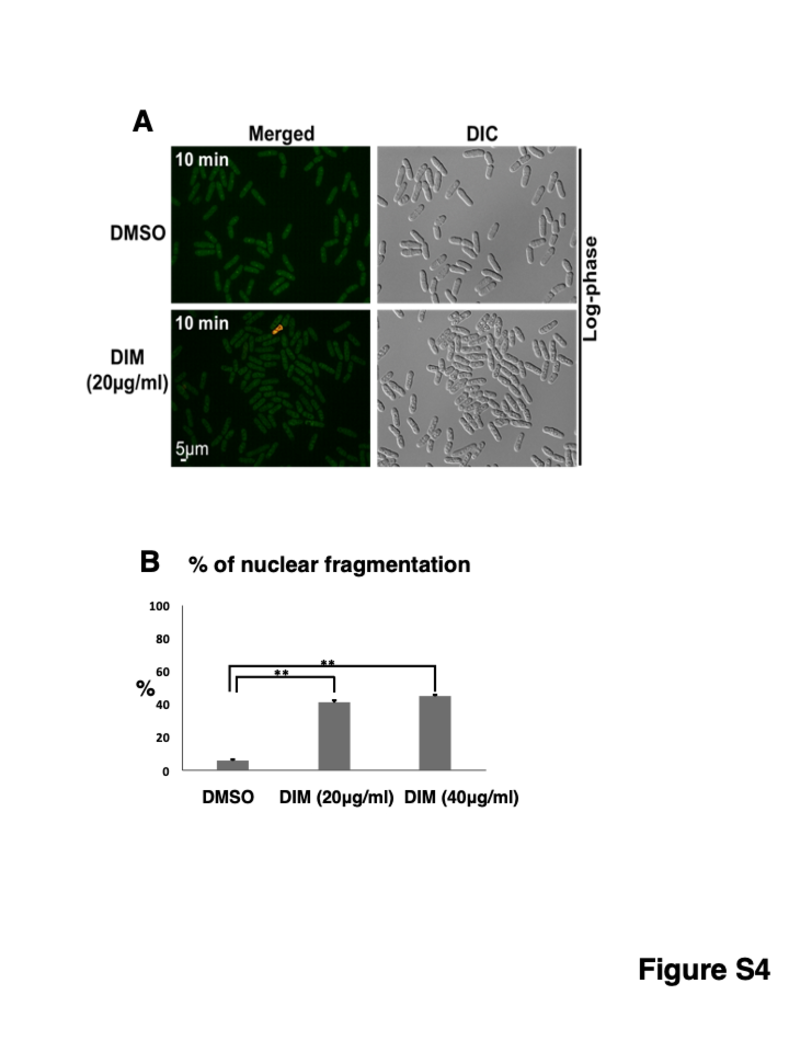

Supplement: S4 Fig — (A) The wild type log-phase cells were treated with 20 μg/ml DIM for 10 minutes. Dead cell abundance was detected by acridine orange (AO)/ ethidium bromide (EB) method as shown in orange color. The percentages of nuclear fragmentation are shown in B. The log phase cells are treated with 20 μg/ml or 40 μg/ml 3,3’-Diindolylmethane (DIM) for 10 minutes. After treatment, cells were washed, precipitated, and transferred to the sample tubes containing sold YEA medium. Six hours later, nuclear fragmentation was monitored by DAPI staining. At least 200 cells were counted with three independent experiments. (TIF) [file pone.0255758.s004.tif]
